# Size of Parks

**Published:** 1860-07

**Authors:** 


It may be of general interest to append the size of the city parks, fractions omitted, and the largest ones elsewhere, premising that the Central Park is, we believe, the largest in the world within the limits of a great city and set apart exclusively for purposes of public pleasure, recreation, and amusement.

| | Acres. | | Acres. |
|---|---|---|---|
| Bowling-Green, New-York, | ½ | St. James', London, | 290 |
| Grammercy, | 1½ | Victoria, | 290 |
| Union Square, | 3 | Regent's, | 360 |
| Stuyvesant, | 4 | Hyde Park, | 395 |
| Hudson, | 4 | Birkenhead, Liverpool, | 500 |
| Madison, | 7 | English Garten, Munich, | 500 |
| Park, City Hall, | 11 | Central Park, New-York, | 844 |
| Battery, (enlarged,) | 25 | Phœnix Park, Dublin, | 1000 |
| Boston Common, (near,) | 50 | Prater, Vienna, | 1500 |
| Fairmount, Philadelphia, | 72 | Bois de Boulogne, near Paris, | 2158 |
| Thiergarten, Berlin, | 200 | Versailles Garden, | 3000 |

